# Do Metacognitions of Children and Adolescents with Anxiety Disorders Change after Intensified Exposure Therapy?

**DOI:** 10.3390/children9020168

**Published:** 2022-01-28

**Authors:** Laura Marie Köcher, Verena Pflug, Silvia Schneider, Hanna Christiansen

**Affiliations:** 1 Clinical Child and Adolescent Psychology, Department of Psychology, Philipps-Universität Marburg, 35032 Marburg, Germany; hanna.christiansen@staff.uni-marburg.de; 2 Mental Health Research and Treatment Center, Ruhr-Universität Bochum, 44787 Bochum, Germany; verena.pflug@rub.de (V.P.); silvia.schneider@rub.de (S.S.)

**Keywords:** anxiety disorder, children, adolescents, metacognition, cognitive behavior therapy, exposure

## Abstract

Metacognitive beliefs have repeatedly proven to play a role in anxiety disorders in children and adolescents, but few studies have investigated whether they change after cognitive behavioral therapy. This longitudinal intervention study explores whether positive and negative metacognitive beliefs in particular change after exposure-focused treatment, and if metacognitive changes predict reductions in anxiety symptoms. A sample of 27 children between 8 and 16 years of age with a primary diagnosis of specific phobia, separation-anxiety disorder or social phobia completed assessments of anxiety symptoms, metacognitive beliefs, worry and repetitive negative thoughts before and after 11 sessions of intensified exposure treatment. Metacognitive beliefs did not change significantly after intensified exposure, but post-hoc power analysis revealed a lack of power here. Change in negative metacognitive beliefs correlated with a change in anxiety symptoms, but did not independently contribute as a predictor variable. Differences between subsamples showed that patients with separation-anxiety disorder scored higher on negative metacognitive beliefs than those with specific or social phobia. Consideration of metacognition, and negative metacognitive beliefs in particular could help us further improve the understanding and treatment of anxiety disorders in children and adolescents and should therefore receive more attention in psychotherapy research.

## 1. Introduction

### 1.1. Treatment of Anxiety Disorders in Children and Adolescents

With a point prevalence of about 10%, anxiety disorders are the most common mental disorders of childhood and adolescence, outpacing depression and hyperkinetic disorders [1]. A recent meta-analysis showed that these numbers have risen even more since the Covid-19 pandemic began, and that every fifth child now exhibits clinically relevant symptoms of an anxiety disorder [2]. The most prevalent anxiety disorder in children and adolescents is specific phobia (5.4–5.6%), followed by separation anxiety disorder (2.0–3.2%) and social phobia (0.1–3.7%) [1,3]. A majority of affected children and adolescents also have comorbid disorders such affective disorders and externalizing disorders [1,4]. Anxiety disorders often persist into adulthood [5], and often go along with later development of mental disorders such as other anxiety disorders, depression or substance use disorders [6,7], more problems in the family, more chronic stress, and less life satisfaction [7].

Despite the large number of children affected, only 13–29% seek psychological help [8]. The current gold standard of therapy for children with anxiety disorders is cognitive behavioral therapy (CBT), as recommended by the clinical guidelines of the National Institute for Health and Care Excellence (NICE) [9] for treatment of social anxiety disorder in children and adolescents. Meta-analyses showed large reductions in anxiety symptoms directly after CBT as well as in follow-up measurements [10,11,12]. These results clearly demonstrate that CBT is an effective psychotherapeutic treatment for various anxiety disorders in children and adolescents. Nevertheless, remission rates appear to differ markedly between individual disorders. For instance, a recent meta-analysis demonstrated that recovery rates after CBT for social phobia (35%) were significantly lower than rates for other anxiety disorders (54%) [13]. Thus, although CBT provides an effective form of treating children and adolescents with anxiety, we need to better understand the mechanisms by which anxiety changes in children and adolescents in order to improve treatment and better tailor it to individual disorder groups.

### 1.2. Negative Repetitive Thoughts in the Context of Anxiety

The influence of one’s thoughts is a relevant topic in this context. There is evidence that repetitive negative thoughts are associated with anxiety [14]. Such cognitions about problems or experiences are characterized by the fact that they occur repeatedly, are (partly) negatively evaluated, and perceived as uncontrollable or engaging [15,16]. Repetitive negative thoughts were examined as a transdiagnostic construct in children and adolescents and are associated with anxiety across different studies [14,17,18]. Repetitive negative thinking in form of excessive worry about various topics is the core criterion of generalized anxiety disorder (GAD), and corresponding worries about specific topics also play a role in separation anxiety disorder or social phobia [19,20,21]. Worry can be defined as future-oriented thoughts about how to deal with potential dangers or challenges [22]. Such thoughts are frequently experienced by children in everyday life [23] but can become problematic if they are experienced more intensively and are perceived as uncontrollable [19]. Studies showed that adolescents with anxiety disorders generally report more worries than non-clinical controls [24,25]. While young patients with GAS and social phobia do not seem to differ [26,27], they report more worry content than children with separation anxiety [26]. Other negative repetitive thoughts in form of pre- and post-event processing have also been associated with social anxiety in children and adolescents [28,29], and socially anxious children and adolescent report significantly more post-event processing than healthy controls [30,31]. After CBT treatment for social phobia, negative post-event processing [31] and worry [32] decreased significantly, although this group effect was only evident for worry when compared to a wait-list control group. This raises the question of whether classical CBT sufficiently addresses all relevant repetitive thoughts.

### 1.3. Metacognitions and Their Role in Psychotherapy

In addition to a purely cognitive focus, metacognitive mechanisms have been increasingly investigated in clinical research over recent decades. Metacognition represents higher-level “cognition about cognitive phenomena” [33] (p. 906), that serve to monitor, control, and evaluate cognitions. Wells and Matthews [34] considered these metacognitive processes in their Self-Regulatory Executive Function (S-REF) model to explain the development and maintenance of mental disorders. They hypothesized that a certain negative thinking style, the Cognitive Attentional Syndrome (CAS), characterizes mental disorders. CAS includes, for example, repetitive negative thoughts such as worry or rumination, as well as selective attention to danger, and maladaptive coping strategies (e.g., thought suppression or avoidance). When CAS is activated, low-level processing is distorted, which in turn reinforces the CAS while affecting the meta-system, which includes the different metacognitive functions and again impacts one’s thinking style. Wells [35] especially assumes that stable schemes about one’s own thoughts in form of positive and negative metacognitive beliefs are relevant to developing and maintaining anxiety disorders: positive metacognitive beliefs evaluate individual thoughts as helpful or singular (e.g., “Worrying helps me cope” [35] (p. 532)). Negative metacognitive beliefs, on the other hand, deal with assumptions about undesirable consequences or the dangers of one’s thoughts (e.g., “Worry could make me lose my mind” [35] (p. 532)). In this regard, negative metacognitive beliefs are attributed as the central role in the development of anxiety disorders. Positive metacognitive beliefs, on the other hand, play a minor role here according to Wells [36].

Multiple studies have looked at the relationship between metacognitive believes and anxiety disorders in children and adolescents: a meta-analysis revealed large overall correlations between negative metacognitive beliefs with anxiety symptoms and worry, whereas these correlations for positive metacognitive beliefs appeared in the range of small to medium effect sizes [37]. Studies comparing patients with anxiety disorders and non-clinical control subjects also paint an ambiguous picture. While some studies only reported higher values for negative metacognitive beliefs in patients with anxiety disorders compared to non-clinical controls, i.e., refs. [27,38,39], others showed both more negative and positive metacognitive beliefs in anxiety-disorder-groups, i.e., refs. [25,40,41]. So far, few studies have compared metacognitive beliefs of different anxiety disorder groups with each other. Bacow [26] found no significant group differences in either positive or negative metacognitive beliefs between patients with separation anxiety, social phobia, obsessive-compulsive disorder, and non-clinical controls. Ellis and Hudson [25] and Hearn et al. [27] also did not demonstrate any significant differences between patients with GAD and social phobia/other anxiety disorders. Esbjørn et al. [42], on the other hand, found that patients with GAD scored higher on negative metacognitive beliefs than those with primary separation anxiety disorder/specific or social phobia, while this difference did not become evident with positive metacognitive beliefs.

With metacognitive therapy (MCT) [43], a transdiagnostic therapy form attempts to change metacognitive beliefs that maintain the symptomatology of mental disorders [44]. In adults, MCT showed promising results treating GAD, social phobia, and other anxiety disorders, e.g., refs. [45,46,47]. A study by Hoffard et al. [48] also showed reductions in positive and negative metacognitive beliefs following CBT and MCT treatment, but a larger decrease after MCT in direct comparison. Other studies support the result that CBT for patients with social phobia leads to change in negative, but not positive metacognitive beliefs [49,50]. For children and adolescents, Simons [51] adapted a Manual by Wells [43] for treating children and adolescents with anxiety disorders, depression, post-traumatic stress disorder and obsessive-compulsive disorder. Esbjørn et al. demonstrated large effects in terms of anxiety symptoms and worries, as well as comparatively high recovery rates of 86–75% for MCT in a sample of 44 7- to 13-year-olds [52]. One study compared MCT and CBT in 7-to-14-year-old patients with GAD and showed large effects for MCT, with no significant differences between interventions [53].

Very few studies involving children and adolescents have examined whether metacognitions change with CBT in those with anxiety disorders. Normann et al. [54] treated 44 children aged 7–12 years with GAS, separation anxiety, social phobia, or specific phobia for 14 weeks with standard CBT techniques that included cognitive restructuring and anxiety confrontations. Metacognitions themselves were not directly processed in therapy. They demonstrated significant reductions on a global measure for metacognitions both after therapy and at 6-month follow-up with medium to large effect sizes. Changes in metacognitions and repetitive negative thoughts were related to changes in child-reported anxiety and significantly predicted these at posttest and follow-up. Hearn et al. [32] examined positive and negative metacognitive beliefs separately in a sample of patients with social phobia aged 8–17 and did not detect changes in positive or negative metacognitive beliefs after CBT, but significant reductions between pretest and 6-months-follow-up. In a randomized controlled trial by Holmes et al. [55] in which patients with GAD underwent GAD-specific CBT in a group setting, no changes in positive metacognitive beliefs appeared between pre-test, post-test and 3-month follow-up, while negative metacognitive beliefs decreased to post-test and follow-up in the therapy group, but did not differ from a waiting control group in pre-post comparison.

### 1.4. Research Questions and Hypothesis

In the current study, we intend to explore how metacognitions change after CBT in more detail, and aim to replicate the findings of Normann et al. [54] specifically for positive and negative metacognitive beliefs. To more precisely describe specific CBT techniques, we investigate whether changes in metacognitions can be initiated by employing a highly controlled, exposure-focused treatment with minimally applied cognitive interventions. We also aim to distinguish the role of global transdiagnostic repetitive negative thinking and worry in order to address specific cognitive mechanisms, and investigate whether patients with specific phobia, separation anxiety disorder, and social phobia differ to see whether the exposure treatment’s different mechanisms take effect. To do this, we address the research questions and hypothesis below:

1. Do repetitive negative thoughts, worry, and metacognitive beliefs of children with anxiety disorders change across treatment with exposure-focused therapy?

Consistent with Normann et al.’s [54] findings to be replicated, we hypothesized that not only anxiety symptoms but also repetitive negative thoughts and metacognitive beliefs would be reduced and that worry also decreases due to the connection to the latter.

2. Are changes in repetitive negative thinking, worry, and anxiety associated with changes in metacognitive beliefs in children with anxiety disorders?

We assume that, in accordance with Normann’s study [54], corresponding positive correlations will also be found in our investigation.

3. Do patients with specific phobia, separation anxiety disorder, and social phobia differ in metacognitive beliefs and in how their metacognitive beliefs change?

Due to the insufficient number of studies so far, we will investigate research question 3 in an exploratory way.

## 2. Materials and Methods

### 2.1. Participants and Procedure

The present study relied on data from the questionnaire study “My Worries-Your Worries”, conducted between 2015 and 2020 at the Child and Adolescent Psychotherapy Outpatient Clinic of the Philipps-University of Marburg. The My Worries-Your Worries study was approved by the Philipps University Marburg’s Ethics Commission (reference number: 2014-47k, 2 September 2014; 2016-34k, 9 November 2016), and the use of data from the Outpatient Clinic for Child and Adolescent Psychotherapy was approved by the Ruhr University Bochum’s Ethics Commission (reference number: 228, 29 September 2015). Non-clinical and clinical samples were surveyed. This study refers to data from the clinical sample only; the non-clinical sample’s data will be published elsewhere. The clinical sample’s children and adolescents were originally recruited for the “KibA-study” at the Outpatient Clinic for Child and Adolescent Psychotherapy Marburg and have participated in study therapy. The KibA-study is a multicenter, randomized-controlled study comparing intensified exposure therapy with and without parental involvement in children and adolescents with anxiety disorders (for details, see German clinical trials register, ID: DRKS00009709, www.drks.de, accessed on 27 January 2022). The high-frequent manualized CBT-treatment consisted of 11 sessions (seven 50-min-sessions and four 100-min-sessions) over seven weeks’ duration. It included psychoeducation, introducing the rationale for exposure, developing self-efficacy-enhancing thoughts, helpful behavior, and coping strategies in anxiety-associated situations, intensified in-vivo-exposure and/or behavioral experiments and relapse prevention. The first session provided children with information on anxiety in general and typical childhood anxieties, features of pathological anxiety and the prevalence of anxiety disorders in children. The specific diagnosis of anxiety diagnosis (separation-anxiety disorder, social phobia, specific phobia) was explained to children and individual anxiety-related situations were collected. In the second to fourth sessions, the connection between thoughts, body and behavior in anxiety was introduced, and self-efficacy-enhancing thoughts and helpful behavior in anxiety situations and attention control were trained. The rationale of exposure therapy is explained in a child-friendly manner, and exposure exercises are planned using an anxiety hierarchy and reward plan. This was followed by four double sessions and one additional session (sessions five to nine) in which exposure exercises were performed in-vivo, high frequently, and in a variety of contexts. The exercises were debriefed with the patients by graphing the child’s progression of anxiety felt during the exercise to clarify the learning experience and document the habituation process. Negative expectations written down before the exercise were checked to see if they had actually occurred, in order to address expectation violations. In this phase, patients also performed exposure exercises at home to ensure the transfer of exposures into everyday life. Sessions 10 and 11 concluded treatment with relapse prevention, in which therapy content was reviewed, visually recorded (i.e., in form of a video), and applied to dealing with future potentially challenging situations. In case of parent involvement, a caregiver/parent attended each session and provided co-therapeutic support for implementing the exposures in the session and at home. Without parental involvement, a caregiver/parent meeting took place after the first session to explain the therapy’s framework, the reward plan, and the implementation of exposures at home without co-therapeutically training the caregiver/parent. Assignment to therapy arms with or without parental involvement was randomized. General results of KibA-study and results on the effect of caregiver/parent-involvement on treatment outcomes will be published elsewhere.

**Figure 1:**
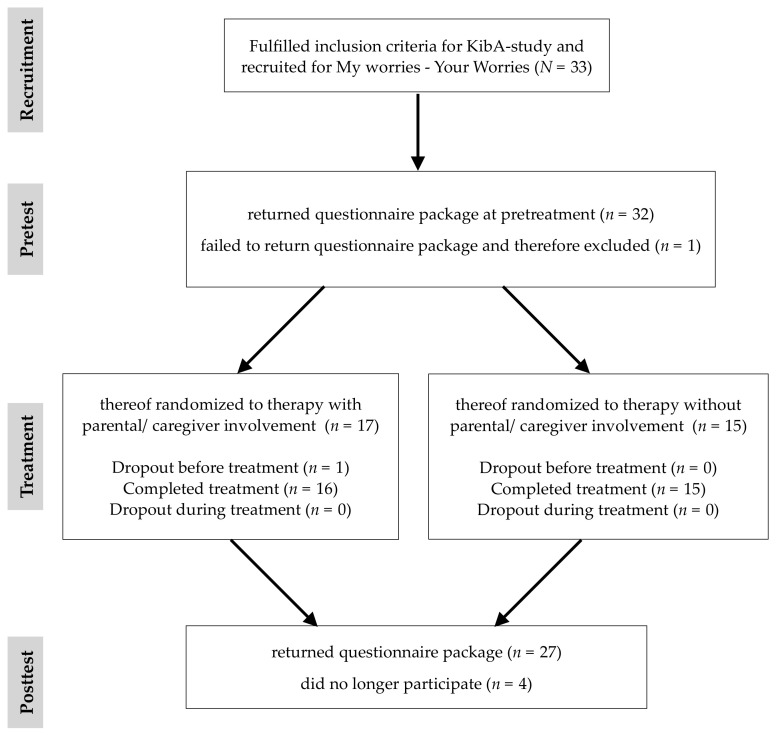
Study Flow diagram.

All families contacted the outpatient clinic themselves and were included if the child was 8–16 years old and fulfilled the diagnostic criteria of social phobia, separation anxiety disorder or specific phobia as a primary diagnosis according to the Diagnostic and Statistical Manual of Mental Disorders, 5th edition (DSM-5) [20] assessed with the Structured Diagnostic Interview for Mental Disorders in Children (Kinder-DIPS) [56]. Families were excluded if the child had been primarily diagnosed with another mental disorder. If the KibA inclusion criteria were met, families were asked if they also wanted to participate in the My Worries-Your Worries study as an add-on research question. If families wanted to participate, children and caregivers/parents were informed about the study in an information letter, and written informed consent for participation was obtained from caregivers/parents and from those children 12 years of age or older. Questionnaire-packages were filled out before and after CBT-treatment by the participants at home, and then returned via post or handed directly to the research-team. Overall, *N* = 33 clinically anxious children and their caregiver/parent were recruited to participate in My Worries-Your Worries, and *n* = 32 returned the questionnaire package before treatment. After treatment, *n* = 27 families filled out the questionnaire package. Overall, 5 families did not participate at posttest. Of those, *n* = 1 dropped out during the treatment completely, *n* = 4 no longer wanted to participate in the study for posttest (see Figure 1).

### 2.2. Measures

#### 2.2.1. Structured Diagnostic Interview for Mental Disorders in Children (Kinder-DIPS)

Primary and co-morbid mental disorders according to DSM-5 were assessed using the original German version of the Kinder-DIPS [56]. Both child- and caregiver/parent versions were carried out separately by trained clinical psychologists. Children were diagnosed provided the DSM-5 criteria for mental disorder were fulfilled by the child and/or caregiver/parent. Despite anxiety disorders, Kinder-DIPS also assesses other important mental disorders such as i.e., attention deficit hyperactivity disorder, conduct disorder, tic disorders, obsessive-compulsive disorder, post-traumatic stress disorder, affective disorders and eating disorders. For an overview of Kinder-DIPS and psychometric quality, see Margraf et al. [57]. Kinder-DIPS shows high interrater-reliability [58] and high satisfaction and acceptance among children and parents [59].

#### 2.2.2. Demographic and Socioeconomic Variables

Age, gender, and school type were addressed with one item each. Participants answered the four items in the Family Affluence Scale II (FAS-II) [60] assessing socio-economic status. Children were asked if they have their own room and if their family owns a car, owns a computer, and has been on vacation the past year. The answer format was simplified to a dichotomous scale (1 = Yes, 0 = No). The values were added up and assigned to three possible levels of family wealth (≤2 = low, 3 = medium, 4 = high). Results are illustrated in Table 1.

#### 2.2.3. Questionnaires for Anxiety- and Obsessive-Compulsive-Disorders (ANZ) of the Diagnostiksystem für Psychische Störungen nach ICD-10 und DSM-IV für Kinder und Jugendliche 2. Version (DISYPS-II)

Symptom level of anxiety was assessed with ANZ [61] in self-report and parent-report format. Both consist of 33 items on a four-point Likert scale ranging from 0 (not at all) to 3 (especially) and have a total scale as well as four subscales (separation anxiety, generalized anxiety, social phobia and specific phobia) measuring anxiety disorders in accordance with DSM-IV. Good Cronbach’s alpha values for the overall scale have been reported for children and adolescents between 11 and 18 years of age and adolescent clinical samples [61,62]. In the current sample, values for Cronbach’s alpha of total-scale ranged between 0.877 (pretest) and 0.914 (posttest) for child-assessment. For caregiver/parent-assessment, Cronbach’s alpha of total-scale was 0.780 for pre- and posttest.

#### 2.2.4. Measure of Excessive Worry Content (EWC)

For a self-report measure of excessive worry content, we applied EWC, a scale adapted in a thesis by Piepenbreier [63] based on an approach described by Bacow et al. [24,26]. The original scale by Bacow et al. [24,26] is based on eight content areas in the Anxiety Disorders Interview Schedule, Child Version (ADIS-IV-C) [64] to measure the severity of worry domains (family, school, every day events, social/interpersonal, health/self, health/others, current events; i.e., “How severe are your fears/worries about your own health?”) in order to address the special features assigned to worry in the metacognitive model [35,36]. The advantage of this method is that it does not just measure the frequency of worries like the Penn State Worry Questionnaire for Children [65]—it also addresses worry content. Piepenbreier [63] used the content areas of Kinder-DIPS GAD-section as a German measure and included the following worry-domains: family, school/sport achievement, everyday events, friends/social, health/self, health/others, current events. Children and adolescents were asked to rate these seven items on a five-point Likert scale ranging from 0 (no worries at all) to 4 (very severe worries). Participants could add other worries in an optional open-ended item. Cronbach’s alphas in our sample for pre- and posttest were 0.777 and 0.824, respectively.

#### 2.2.5. Perseverative Thinking Questionnaire (PTQ)

Dysfunctional and repetitive negative thoughts were assessed by applying the self-report questionnaire PTQ [16]. Its scale consists of 15 items (i.e., “I think of many problems without solving any of them”) rated on a five-point Likert scale ranging from 0 (never) to 4 (almost all the time). Ehring et al. [66] demonstrated its adequate reliability and validity for adult samples. The questionnaire was administered by De Voogd et al. [67] in students aged between 11 and 19 years and revealed internal consistency of α = 0.95. In the present sample, Cronbach’s alpha was 0.944 at pretest and 0.950 at posttest.

#### 2.2.6. German Metacognitions Questionnaire for Children (MKF-K)

The MKF-K [68] was used to measure metacognitive beliefs. It was constructed based on the Metacognition Questionnaire-short version (MKF-30) [69], which is a German translation of the Metacognition Questionnaire-30-item version (MCQ-30) [70]. MKF-K consists of 30 items and uses a four-point Likert scale of 0 (not at all) to 3 (completely). It contains a total scale and four subscales: positive beliefs, negative beliefs, cognitive confidence, and cognitive self-consciousness. In the current study, we only used positive beliefs (POS, i.e., “When I worry, I can think more clearly”) and negative beliefs (NEG, i.e., “If I keep worrying, I may go crazy”) subscales and for comparison to Normann et al. [54] also report results for the total scale. The internal consistency of the overall scale (α = 0.76) and the subscales (α = 0.70 to 0.77) in a sample of 7-to-14-year-old children can be classified as satisfactory, and large correlations with worry and anxiety-symptoms speak for convergent validity [68]. In the present sample, Cronbach’s alpha ranged from 0.841 (pretest) to 0.867 (posttest) for the total scale, from 0.732 (pretest) to 0.809 (posttest) for positive metacognitive beliefs and from 0.582 (pretest) to 0.452 (posttest) for negative metacognitive beliefs.

### 2.3. Statistical Analysis

Statistical analysis was carried out with IBM SPSS Statistics 27. For post-hoc-power-analysis, we used the program G*Power 3.1 [71]. First, we analyzed if the demographic variables differed between the diagnosis subgroups via χ^2^-tests for gender, school type and randomization to therapy with or without caregiver/parent-involvement. Because of not normally distributed data, we used Kruskall-Wallis-tests for age, FAS-II scores and number of comorbidities. Second, we examined mean-differences from the pre- to posttest with repeated measures *t*-tests, and calculated Cohen’s *d* as effect size measure for all anxiety symptoms, internalizing symptoms, worry, repetitive negative thinking and metacognitions. Conventions of Cohen [72] indicate that values of *d* = 0.8 refers to large, *d* = 0.5 to medium, and *d* = 0.2 to small effect sizes. Because of multiple testing, we adjusted the alpha-level using the Bonferroni correction. In case of not normally distributed data, we used the nonparametric Bootstrapping procedure with 10,000 iterations to gain large power [73]. Third, we calculated change scores (pretest-score-posttest-score) for all outcomes, and afterwards performed Pearson’s product-moment-correlations between all change-scores. Effect sizes of *r* = 0.1 were interpreted as small, *r* = 0.3 as medium and *r* = 0.5 as large as suggested by Cohen [72]. Fourth, we conducted a regression analysis on change-scores of child-reported anxiety-symptoms. Significantly correlating change-scores with this outcome were entered simultaneously as predictor variables in order to explore whether POS and NEG are unique predictors for reductions in anxiety symptoms after controlling for other correlating constructs. Fifth, we exploratively examined whether patients with social phobia, patients with separation anxiety disorder and those with specific phobia differed in outcome variables. Because of our small and unequally distributed subsamples, we tested for group differences nonparametrically using exact Kruskal-Wallis tests. Because of the subgroups’ small sample sizes (especially the social-phobia group’s), we conducted exact tests as recommended by Meyer and Seaman [74] instead of asymptotic χ^2^ approximations. Sixth, we determined the power of the non-significant results. To estimate the power of Kruskall-Wallis-tests, power estimation methods are reported to be accurate and reliable only if subsample sizes are equal and each greater than *n* = 10 or 100, respectively [75,76]. Since our group comparisons do not meet these requirements and simulation studies have shown that the power tends to be greater than that of a corresponding ANOVA [77,78], the power of the one-factor ANOVA was analyzed here as an approximation.

## 3. Results

Results for demographic variables are displayed in Table 1. One participant was excluded from further analysis due to extreme values. Our final sample consisted of *n* = 31 participants, *n* = 26 from whom we had posttreatment measures available. The total sample consisted of 16 girls and 15 boys. Mean age was 10.36 (*SD* = 2.17), range 8–16 years. Overall, 38.7% (*n* = 12) of children were diagnosed with separation-anxiety disorder, 38.7% (*n* = 12) with specific phobia, and 22.6% (*n* = 7) with social phobia. A total of 32.3% (*n* = 10) of children were diagnosed with one and 35.4% (*n* = 11) with two or more comorbid disorders. One child with primary specific phobia had a comorbid GAD diagnosis. 30 children filled out information about their social-economic-status in FAS; most (64.5%) of the children live in a wealthy family while 19.4% of children’s families were of moderate and 12.9% low wealth.

In terms of school type, almost half of the sample goes to elementary school (48.4%), while 51.6% visit different types of secondary schools (25.8% grammar school; 9.7%, secondary school; 12.9% comprehensive secondary schools; 3.2% schools for special needs). Participating caregivers were mothers in *n* = 25 cases and fathers in *n* = 4 cases, *n* = 2 made no specification in this regard. Diagnostic groups did not differ significantly in age (χ^2^ (2) = 1.559, *p* = 0.459), gender (χ^2^ (2) = 1.588, *p* = 0.452), school type (χ^2^ (8) = 6.369, *p* = 0.606), FAS-II mean score (χ*^2^* (2) = 0.022, *p* = 0.989), number of comorbidities (χ^2^ (2) = 0.820, *p* = 0.664) or randomization (χ^2^ (2) = 1.588, *p* = 0.452).

### 3.1. Changes across Time

Mean scores, standard deviations for outcome measures, and results for test statistic and effect sizes for anxiety symptoms, internalizing symptoms, worry, repetitive negative thoughts and metacognitions for pre- and posttest are displayed in Table 2. Because values for anxiety symptoms (self-assessment), POS and NEG were not normally distributed, we used the bootstrapping method with 10,000 iterations. We performed Bonferroni correction and used an adjusted alpha-level of 0.007. *t*-tests revealed significant reductions from pre- to posttest for anxiety symptoms in self- and caregiver/parent-assessment as well as for worry (*p* < 0.006). Effect sizes speak for large effects for anxiety and a medium effect for worry reductions. No significant changes appeared in repetitive negative thoughts or metacognitive beliefs.

### 3.2. Relationships between Changes in Anxiety, Worry and Metacognitions

Correlations between outcome measures’ change-scores are illustrated in Table 3. The MKF-total correlated with all variables with medium to large effect sizes, while the NEG correlated significantly with child-reported anxiety, caregiver/parent-reported anxiety, and negative thinking with medium to large effect sizes. POS did not correlate with other changes in outcomes despite the MKF-total, and was therefore excluded from regression analysis.

### 3.3. Regression Analysis

We analyzed whether significant correlations with change-scores in child reported anxiety symptoms contributed independently to alleviating child-reported anxiety symptoms via regression analysis. Results are displayed in Table 4. In the MFK’s case, we only included the NEG-subscale and not the MKF-total, since the latter also contains items from this subscale. The overall model for change in child-reported anxiety symptoms was significant, *F*(3, 21) = 11.554, *p* < 0.001 and showed a high amount of explained variance (*R*^2^ = 0.623, corrected *R*^2^ = 0.569) according to Cohen [79]. Change in worry proved to be a significant predictor variable. We observed no significant contribution regarding change in child-reported anxiety symptoms concerning any change in repetitive negative thinking or in NEG.

### 3.4. Exploratory Comparison of the Diagnostic Groups

To test differences in central tendencies in diagnostic groups exploratively, we ran exact non-parametric Kruskal-Wallis tests. We noted differences in NEG at pretest (χ^2^ (2) = 9.431, *p* = 0.006) regarding the groups’ central tendencies. Post-hoc Dunn-Bonferroni-tests showed that the separation-anxiety disorder group had significantly higher scores in NEG than the specific phobia group (*z* = 2.939, *p* = 0.003) and social phobia group (*z* = 2.066, *p* = 0.039), while the latter groups did not differ (*z* = 0.457, *p* = 0.648). There was no significant difference in the NEG change scores. For the other outcome variables (anxiety symptoms in self- and caregiver assessment, worry, repetitive negative thoughts, POS), the groups’ central tendencies did not differ significantly in either the pretest- or change-scores.

### 3.5. Post-Hoc Power Analysis

Cohen [72] recommends a 1−β = 0.80 test strength for statistical analysis. For our non-significant *t*-tests of change in metacognitions and repetitive negative thinking we achieved a non-satisfactory power of 1−β = 0.403 for POS, 1−β = 0.287 for NEG, 1−β = 0.191 for MKF-total score and 1−β = 0.604 for PTQ. Power for non-significant tests of group differences in demographic variables with Kruskall-Wallis-tests (1−β = 0.082–0.199) and χ^2^-tests showed also non-satisfactory power (1−β = 0.186–0.613). Power ranged between 1−β = 0.054–0.24 for non-significant differences in pre-test scores and for change-scores between 1−β = 0.065–0.397.

## 4. Discussion

### 4.1. Change in Anxiety, Worry and Repetitive Negative Thoughts

This study investigated whether repetitive negative thinking, worry, and metacognitive beliefs change through exposure-focused therapy in children and adolescents with specific phobia, separation-anxiety disorder and social phobia, and if these changes contribute to treatment benefits in reducing anxiety.

In both the self-assessment and caregiver-assessment, we observed a reduction in anxiety symptoms in the large-effects range after exposure-focused therapy. These effects can be compared with the results of a meta-analysis by In-Albon and Schneider [10] on anxiety reduction in CBT interventions in children and adolescents. As expected, patients’ excessive worry lessened from pretest to posttest with a medium effect size. We demonstrate that even though it was not directly targeted during treatment, worry can be changed through exposure. Changes in worry significantly correlated with and predicted anxiety symptom changes in self-assessment. Interestingly, we did not observe this correlation regarding repetitive negative thoughts. Here, using Bonferroni correction for alpha-level, no significant decrease from pretest to posttest appeared. Moreover, change in repetitive negative thinking did not explain the variance in changes in anxiety after controlling for worry. These results underscore the importance of worry not only in the GAD context, but also regarding specific phobia, separation-anxiety disorder, and social phobia.

### 4.2. Change in Metacognitive Beliefs

Contrary to our hypothesis, we also did not find any significant changes from pre- to post-test in POS or NEG through exposure-focused therapy. Our results thus do not support the treatment gains in metacognitions reported by Normann et al. [54]; however, post-hoc power analysis showed that our study was underpowered to detect possible effects for POS (*d* = 0.218) and NEG (*d* = 0.282) reductions. It is therefore possible that our deviating findings might be attributable to this power problem. In contrast, our results are consistent with Hearn et al. [32], who found significant reductions in worry, but not in POS or NEG, at posttest after the CBT treatment of patients with social phobia. Nevertheless, changes in repetitive negative thinking, NEG and total MKF scores correlated significantly with changes in anxiety symptoms in both self-judgment and caregiver judgment. This concurs with the results of Normann et al. [54], and effect sizes for MKF-total-score correlations with anxiety in self-assessment are comparable. But unlike their study, we demonstrated no metacognitive changes as predictors of anxiety symptom changes. A recently published study by Wolenski et al. [80] tested whether metacognitions measured before CBT therapy predicted anxiety scores after therapy, without controlling for worry’s influence. In their study, neither POS nor NEG were significant predictors, in line with our findings. Moreover, changes in NEG but not POS correlated with change in anxiety symptoms [80]. This too is consistent with our findings, as changes in POS were also unrelated to changes in anxiety and highlight the special role of NEG in contrast to POS that Wells emphasized [36]. Intercorrelations showed that changes in NEG did correlate significantly with changes in anxiety and repetitive negative thinking, even if we could not demonstrate any significant changes in NEG from pre- to posttest in repeated measure *t*-test, which again could be due to underpowered testing.

To the best of our knowledge, only a few working groups have investigated metacognitive changes after CBT for anxiety disorders so far [32,54,55,80]. Some studies on MCT for children and adolescents with anxiety disorders also analyzed metacognitive beliefs as outcome variables. One study showed that both POS and NEG decreased significantly after group-based MCT treatment in a sample of 44 children with a primary GAD-diagnosis [52]. Simons and Vloet [81] examined MCT in three female adolescents diagnosed with emetophobia in their single-case study, and reported a descriptive reduction in POS in one of the three girls, while the values remained stable in the other two. In contrast, a descriptive decrease in NEG was observed in all three patients. In a pilot study by Thorslund et al. [82] ten adolescents with anxiety or depressive disorders were treated via six sessions of MCT in a group-based format. POS was reduced with a small effect from pre-to posttest, with medium effect at 1-month-follow-up and a large effect at 3-month-follow-up, while the reduction in NEG referred to a large effect at each timepoint. This evidence concurs with CBT findings of Hearn et al. [32] and Holmes et al. [55] and suggests that metacognitions appear reduced only at follow-up measurements three months after completing therapy, leaving open whether this is due to a treatment effect that only becomes apparent after time, or a simple time effect [55]. Our study does not reflect this because we conducted no follow-up examinations of metacognitions.

Our treatment included fewer cognitive elements than described in the CBT-Studies by Holmes et al. [55] and Normann et al. [54]. Hearn [32] did not target worry or metacognitive beliefs either directly with their CBT-treatment of social phobia, but they did observe the same result pattern as our study, namely a reduction in worry but not in metacognitive beliefs immediately after the treatment as in our study. It is possible that stronger (meta-)cognitive focus is needed in therapy in addition to exposure elements to induce changes in metacognitions, but it is also possible that potential effects could not be reflected in our study due to the low power, as substantial correlations appeared between symptom reduction and change in NEG. For example, the cognitively-focussed No worries! program administered by Holmes et al. [55] was designed to specifically change NEG and resulted in significant reductions in NEG but not in POS; only reductions in worries differed significantly between their treatment and waitlist control groups.

### 4.3. Comparisons of Diagnostic Subsamples

Exploratory comparison of patients with specific phobia, separation-anxiety disorder and social phobia showed that the separation-anxiety disorder group held significantly higher NEG at pretest than the specific or social phobia groups. To the best of our knowledge, only Bacow [26] has tested whether children with separation anxiety and social phobia differ in POS or NEG so far, and they did not observe any significant group differences. In this respect, our result appears surprising, as worries—which are closely related to metacognitions—are relevant in the context of separation anxiety [19,21], but are known to be significantly higher in patients with social phobia [26]. One possible explanation for this result might be that our separation-anxiety disorder group is descriptively slightly younger than the other groups, and two studies have shown that age is related to NEG with small negative effects [83,84]. However, the majority of studies examining age effects in metacognition have not reported significant results [37], and age differences between groups were not significant here. Subgroups did also not differ significantly in any other of the explored demographic variables or randomization: not in worry, anxiety, or repetitive negative thinking. Their investigation demonstrated no other significant differences before therapy or in any changes through therapy, a finding in line with the results of Bacow et al. [26].

### 4.4. Limitations

To the best of our knowledge, our study is the first to measure metacognitive beliefs in children and adolescents before and after high-frequent exposure-focused treatment. Treatment was manualized and highly controlled. We opted for an exposure-focused intervention in order to make clearer statements about the specific mechanisms involved in exposure treatments, and to be able to control for cognitive change through therapy to the greatest extent possible. We explicitly focused on positive and negative metacognitive beliefs and included a transdiagnostic measure of repetitive negative thinking as well as a measure of worry. Diagnoses were based on a widely used and well evaluated structured clinical interview conducted with children/adolescents and parents/caregivers. The inclusion of children and adolescents with specific phobia, separation-anxiety disorder and social phobia allowed us to compare metacognitive beliefs of diagnostic subsamples and contributes to understanding of metacognitive processes in these disorders.

However, a number of limitations of this study must be considered.

First, our sample size was small (*n* = 31), and post-hoc-power-analysis indicates a lack of power. Non-significant effect sizes for possible mean differences between pre- to posttest in POS and NEG were small, which contrasts with the higher pre-post effects of Cohen’s *d* = 0.55 reported by Normann et al. [54]. Despite the lack of power, this suggests that our study did actually achieve results differing from Normann’s study. For differences in diagnostic subgroups, only a significant result for NEG appeared. We cannot therefore assume that there are no differences at all in change scores and pretest-values of POS, repetitive negative thinking, and worry.

Second, our sample included a high proportion of patients from wealthy families and deviates from Normann et al. [54] in its age and anxiety diagnosis structure. Our sample was older as we also included youths up to 16 years of age to be able to make statements about both children and adolescents. We did not include patients with a GAD-primary diagnosis since most studies deal with GAD, as we deliberately wanted to focus on other disorders to fill the research gap described in this regard, and only one child in our sample presented a comorbid GAD diagnosis. Also, only one third of participants had no comorbid mental disorder, which corresponds to clinical practice in terms of epidemiological data [85] but makes it difficult to make concrete statements comparing the individual disorder groups.

Third, we administered two questionnaires not yet validated across different studies. The measure of excessive worry content was used because it was specifically introduced by Bacow et al. [24,26] to measure worry as defined in the metacognitive model of generalized anxiety disorder [35,36] and was first used in a German adaptation. In the present sample, this questionnaire revealed good internal consistency. The MKF-K is the first metacognitive measure for children and adolescents available in the German language and showed adequate psychometrics and acceptable Cronbach’s alpha of ≥0.70 for all subscales in the study of Naumann [68] for a large representative school sample of 972 children and adolescents between seven and 14 years of age. In the present clinical sample, Cronbach’s alpha for total scale and POS subscale reached comparable to slightly higher values, but the NEG subscale fell under the acceptable range at pretest and posttest. To measure transdiagnostic repetitive negative thinking, we applied the PTQ. It was not adapted for use with children and adolescents but has shown excellent Cronbach’s alpha values in child and adolescent samples [67,86] as well as in the current sample comparable to data on adults [16,66]. A children’s version of the English PTQ has also been available since 2015 [17] but was not available to us while planning this study.

Fourth, we used an exposure-focused intervention, while Normann et al. [54] had included cognitive restructuring in their intervention, so our study’s psychotherapeutic interventions cannot be compared one-to-one here.

Fifth, as we conducted no follow-up assessments, we cannot comment on whether metacognitions as described by Hearn et al. [32] might have changed after completing the intervention. Nevertheless, the pre-post comparison allows us to report that exposure-focused therapy did not lead to a reduction in metacognitive beliefs immediately after therapy, while worry itself was reduced with a medium effect, although neither worry nor metacognitions were specifically targeted during treatment.

## 5. Conclusions

The results of this study only partially support the findings of Normann et al. [54]. We did not demonstrate metacognitive changes following exposure treatment in children and adolescents with anxiety disorders. Since changes in NEG nevertheless did correlate with changes in anxiety symptoms, metacognitions seem to be relevant for the treatment of anxiety disorders, and they represent an additional target in treating anxiety disorders. Before therapy, patients with separation-anxiety disorder held more NEG than patients with specific or social phobia. Overall, it becomes clear that metacognitions play a role not only in generalized anxiety, but also in the context of other anxiety disorders in childhood and youth. Research should focus on investigating metacognitive beliefs in different diagnosis, to allow conclusions about their relevance for specific disorders. To better understand the mechanisms of anxiety reduction, the role of metacognitions should also be further investigated to better optimize therapies for anxiety disorders. Further research with larger samples is needed to investigate the influence of metacognitive changes on reducing anxiety and to analyze differences between different anxiety disorders. To this end, metacognitions should also be examined more frequently in therapy studies in order to draw more conclusions. To examine whether metacognitions change substantially after therapy completion, future studies should include follow-up measures of metacognitions. In this context, the psychometric quality criteria of the MKF-K should also undergo additional investigation. This knowledge could help us improve the care of children and adolescents suffering from pathological anxiety, especially to discover useful approaches when patients do not sufficiently benefit from exposure treatment.

## Figures and Tables

**Table 1:** Demographic variables.

Variable	Total	SAD	SepAD	SP
	*n* = 31	*n* = 7	*n* = 12	*n* = 12
Age, *M* (*SD*)	10.36 (2.17)	10.05 (1.99)	9.84 (1.41)	10.25 (2.49)
Gender, *n* (%) female	16 (51.6)	5 (71.4)	5 (41.7)	6 (50.0)
School type, *n* (%)				
elementary school	15 (48.4)	3 (42.9)	6 (50.0)	6 (50.0)
grammar school	8 (25.8)	1 (14.3)	4 (33.3)	3 (25.0)
comprehensive secondary school	4 (12.9)	1 (14.3)	1 (8.3)	2 (16.7)
secondary school	3 (9.7)	2 (28.6)	-	1 (8.3)
school for special needs	1 (3.2)	-	1 (8.3)	-
FAS-II, *n* (%) ^a^				
High family wealth	20 (64.5)	5 (71.4)	7 (58.3)	8 (66.7)
Moderate family wealth	6 (19.4)	1 (14.3)	3 (25.0)	2 (16.7)
Low family wealth	4 (12.9)	1 (14.3)	1 (8.3)	2 (16.7)
Comorbid disorder, *n (%)*				
None	10 (32.3)	-	4 (33.3)	6 (50.0)
One	10 (32.3)	5 (71.4)	4 (33.3)	1 (8.3)
Two or more	11 (35.4)	2 (28.6)	4 (33.3)	5 (41.7)
Comorbid GAD	1 (3.2)	-	-	1 (8.3)
Randomization, *n* (%)				
With caregiver/parent	16 (51.6)	5 (71.4)	6 (50.0)	5 (41.7)
Without caregiver/parent	15 (48.4)	2 (28.6)	6 (50.0)	7 (58.3)

Note. *M* = Mean, *SD* = Standard division, *n* = sample size, FAS-II = Family Affluence Scale II. SAD = social phobia, SepAD = separation-anxiety disorder, SP = specific phobia, GAD = generalized anxiety disorder. ^a^ *n* = 30.

**Table 2:** Means (standard divisions) and test-statistic on anxiety, metacognitions and repetitive thinking at pretest and posttest.

Measure	Pretest		Posttest		Test Statistic	*d*
	Range	*M* (*SD*)	Range	*M* (*SD*)		
ANZ_child_	3.0–50.0	23.64 (14.62) ^a^	0.0–32.0	9.89 (10.59) ^a^	95%-CI [9.37; 18.16] *^,b^	1.077
ANZ_parent_	3.0–40.0	19.69 (10.26) ^a^	0.0–23.0	8.43 (6.32) ^a^	*t*(24) = 6.295, *p* < 0.001 *	1.321
EWC	0.0–23.0	8.86 (6.27)	0.0–13.0	5.31 (4.14)	*t*(25) = 4.027, *p* < 0.001 *	0.668
PTQ	0.0–49.3	14.65 (11.55)	0.0–38.0	10.35 (10.79)	*t*(25) = 2.256, *p* = 0.017	0.385
MKF-POS	0.0–8.0	1.35 (2.28)	0.0–5.0	0.92 (1.60)	95%-CI [−0.44; 1.40] ^b^	0.218
MKF-NEG	0.0–9.8	2.88 (2.24)	0.0–8.0	2.31 (1.78)	95%-CI [−0.33; 1.59] ^b^	0.282
MKF-total	1.0–38.0	14.90 (9.08)	1.0–33.0	13.49 (9.15)	*t*(25) = 0.791, *p* = 0.219	0.155

Note. *n* = 26. ANZ = Questionnaires for anxiety- and obsessive-compulsive-disorders of DISYPS-II, child = assessment of child/adolescent, parent = assessment of caregiver/parent, EWC = Measure of excessive worry content, PTQ = Perseverative Thinking Questionnaire, MKF = German Metacognitions Questionnaire for Children, POS = positive beliefs about worry, NEG = negative beliefs about worry. * *p* < 0.007, adjusted alpha level. ^a^ *n* = 25, ^b^ Bootstrapping with 10,000 iterations because of non-normally distributed data.

**Table 3:** Intercorrelations among change-scores from pre- to posttest.

Measure	2	3	4	5	6	7
1. Δ ANZ_child_ ^a^	0.304	0.759 ***	0.635 ***	0.182	0.364 *	0.483 **
2. Δ ANZ_parent_ ^a^		0.269	0.433 *	0.209	0.347 *	0.455 *
3. Δ EWC			0.657 ***	0.118	0.219	0.404 *
4. Δ PTQ				0.123	0.485 **	0.602 **
5. Δ MKF-POS					0.163	0.515 **
6. Δ MKF-NEG						0.680 ***
7. Δ MKF-total						

Note. *n* = 26. Δ = change score, ANZ = Questionnaires for anxiety- and obsessive-compulsive-disorders of DISYPS-II, child = assessment of child/adolescent, parent = assessment of caregiver/parent, EWC = Measure of excessive worry content, PTQ = Perseverative Thinking Questionnaire, MKF = German Metacognitions Questionnaire for Children, POS = positive beliefs about worry, NEG = negative beliefs about worry. *** *p* < 0.001, ** *p* < 0.01, * *p* < 0.05. ^a^ *n* = 25.

**Table 4:** Linear Model of predictors for anxiety symptom changes.

Measure	*B*	*SE B*	β	*R* ^2^
Δ ANZ_child_				0.623 **
Δ EWC	1.593	0.457	0.646 *	
Δ PTQ	0.136	0.249	0.115	
Δ MKF-NEG	0.706	0.692	0.163	

Note. *n* = 25. Δ = change score. ANZ = Questionnaires for anxiety- and obsessive-compulsive-disorders of DISYPS-II, child = assessment of child/adolescent, EWC = Measure of excessive worry content, PTQ = Perseverative Thinking Questionnaire, MKF = German Metacognitions Questionnaire for Children, NEG = negative beliefs about worry. * *p* < 0.01; ** *p* < 0.001.

## Data Availability

The data presented in this study are available on request from the corresponding author. The datasets are stored at the University of Marburg and can be accessed there. The data is not publicly available due to the fact that this is not in accordance with consent provided by participants on the use of confidential data.
